# Claudin 18 Immunohistochemistry Expression in Salivary Gland Neoplasms: Limited Potential as a Therapeutic Biomarker

**DOI:** 10.1007/s12105-026-01972-6

**Published:** 2026-09-24

**Authors:** Nicole Fye, Sanica Bhele

**Affiliations:** Department of Pathology and Laboratory Medicine, Penn State Milton S. Hershey Medical Center, 500 University Dr, Hershey, PA 17033 USA

**Keywords:** Claudin 18, Targeted therapy, Salivary gland, Biomarker

## Abstract

**Purpose:**

Claudin 18 (CLDN18) is a protein that functions to maintain the integrity of tight junctions in epithelial cells. Recent development of anti-Claudin 18.2 targeted therapies have yielded positive survival outcomes for patients with Claudin 18 expression in gastric carcinomas. This study aims to investigate whether expression of Claudin 18 is present in a variety of benign and malignant salivary gland neoplasms.

**Methods:**

Forty-eight cases of salivary gland neoplasms of various subtypes were included for this pilot study. Ancillary data including tumor location, grade, and stage of tumor as applicable was collected. Claudin 18 immunohistochemistry was performed on a representative tumor block of each case.

**Results:**

No aberrant Claudin 18 expression was seen in the benign as well as malignant salivary gland neoplasms in this pilot study.

**Conclusions:**

CLDN18 expression was uniformly absent across the tumor types, locations, grades, and stages represented in this cohort. The absence of expression in all cases indicates that Claudin 18 has limited potential as a therapeutic marker in patients with salivary gland neoplasms. This study contributes to the extremely limited literature regarding Claudin 18 expression within salivary gland neoplasms.

## Introduction

Aberrant expression of immunohistochemical markers in cancer increasingly serves not only as a diagnostic clue but also as a therapeutically actionable biomarker. Epigenetic variability influences gene expression and is a widely recognized factor in the formation and progression of cancer. Alterations in DNA methylation, histone modification, and non-coding RNA expression promote the ability of tumors to undergo further differentiation and heterogeneity. While these abnormalities cause challenges in treatment by increasing tumor diversity, they also serve as biomarkers for targeted therapies [1].

Claudin 18 is a protein expressed by the *CLDN18* gene and is responsible for the maintenance of tight junctions in epithelial cells [2]. The CLDN18 gene is located at chromosome 3q22 and is composed of six exons with five interspersed introns. Two protein isoforms are created via alternative splicing: CLDN18.1 and CLDN18.2. CLDN18.1 is primarily expressed in lung tissue. The Claudin 18.2 isotype is expressed in benign gastric epithelium, but its accessibility on the surface of tumor cells during malignant transformation has made it a target for emerging anti-Claudin 18.2 therapies. These new agents have proven beneficial in increasing overall survival and progression-free survival in patients with advanced or unresectable gastric carcinomas [3–5].

The success of anti-biomarker therapy in gastric cancers raises the important question of whether similar expression patterns in other high-risk neoplasms can yield more therapeutic options for these entities. Salivary gland carcinomas are diverse in terms of behavior, and many aggressive subtypes exist. This makes early detection and treatment of these neoplasms imperative for patient outcomes [6].

Current available actionable biomarkers for targeted therapy in salivary gland neoplasms include androgen receptor (AR), Human Epidermal Growth Factor Receptor 2 (HER2), and Neurotrophic Tropomyosin Receptor Kinases (NTRK) [7]. While targeted therapies for these biomarkers have promising outcomes, these are often expressed in limited subsets of salivary gland carcinomas, leaving many tumors ineligible for targeted intervention [7]. Therefore, it is crucial to continue efforts towards molecular profiling and elucidating biomarker expression in salivary gland neoplasms to improve treatment options for patients.

Claudin 18 is expressed in gastric epithelium with glandular and secretory function. We hypothesized that salivary gland neoplasms with a similar glandular nature would express Claudin 18. Particular interest was focused on mucoepidermoid carcinoma, as these neoplasms have previously been shown to express several other claudin proteins [8], and their phenotypic heterogeneity provides an opportunity to evaluate potential variability in Claudin 18 expression across different cellular components. The purpose of this pilot study was to investigate the presence or absence of expression of Claudin 18 in salivary gland neoplasms to determine the potential of Claudin 18 as a biomarker for targeted therapy.

## Materials and Methods

With Institutional Review Board (IRB) approval (protocol number STUDY00027211), cases of salivary gland neoplasms at a single institution from January 2022–January 2026 were queried and stratified by tumor type. All primary salivary gland tumor types were included for analysis. The five most recent consecutive cases of each tumor type were included in this pilot. This approach was chosen to minimize selection bias while prioritizing the use of newer slides, which are expected to have better preservation and quality for immunohistochemical staining. All cases of rarer tumor types in which less than five cases were available during this time period were included. Nine cases of mucoepidermoid carcinoma were included due to its phenotypic variability and the hypothesized high pretest probability of Claudin 18 expression. In total, forty-eight salivary gland tumors were identified for analysis (Table 1). IHC staining was carried out using CLDN18 antibody (clone 43-14A) on a Ventana Discovery Ultra Platform with Optiview Detection Kit (Roche #950–500). This antibody detects both Claudin 18.1 and 18.2 isoforms but effectively reflects the presence of Claudin 18.2 in gastroesophageal cancers given the absence of Claudin 18.1 in these tumor sites [5].

**Table 1:** Number of cases of each type of salivary gland neoplasm included in this study

Salivary gland neoplasm	Number of cases
Basal Cell Adenoma	5
Oncocytoma	5
Pleomorphic Adenoma	5
Warthin Tumor	5
Acinic Cell Carcinoma	5
Adenoid Cystic Carcinoma	5
Carcinoma ex-Pleomorphic Adenoma	3
Mucoepidermoid Carcinoma	9
Polymorphous Adenocarcinoma	1
Salivary Duct Carcinoma	3
Salivary Gland Carcinoma, not otherwise specified	2

Additional information including immunohistochemical staining performed for diagnosis, tumor stage and grade as applicable, and tumor location were collected.

**Fig. 1:**
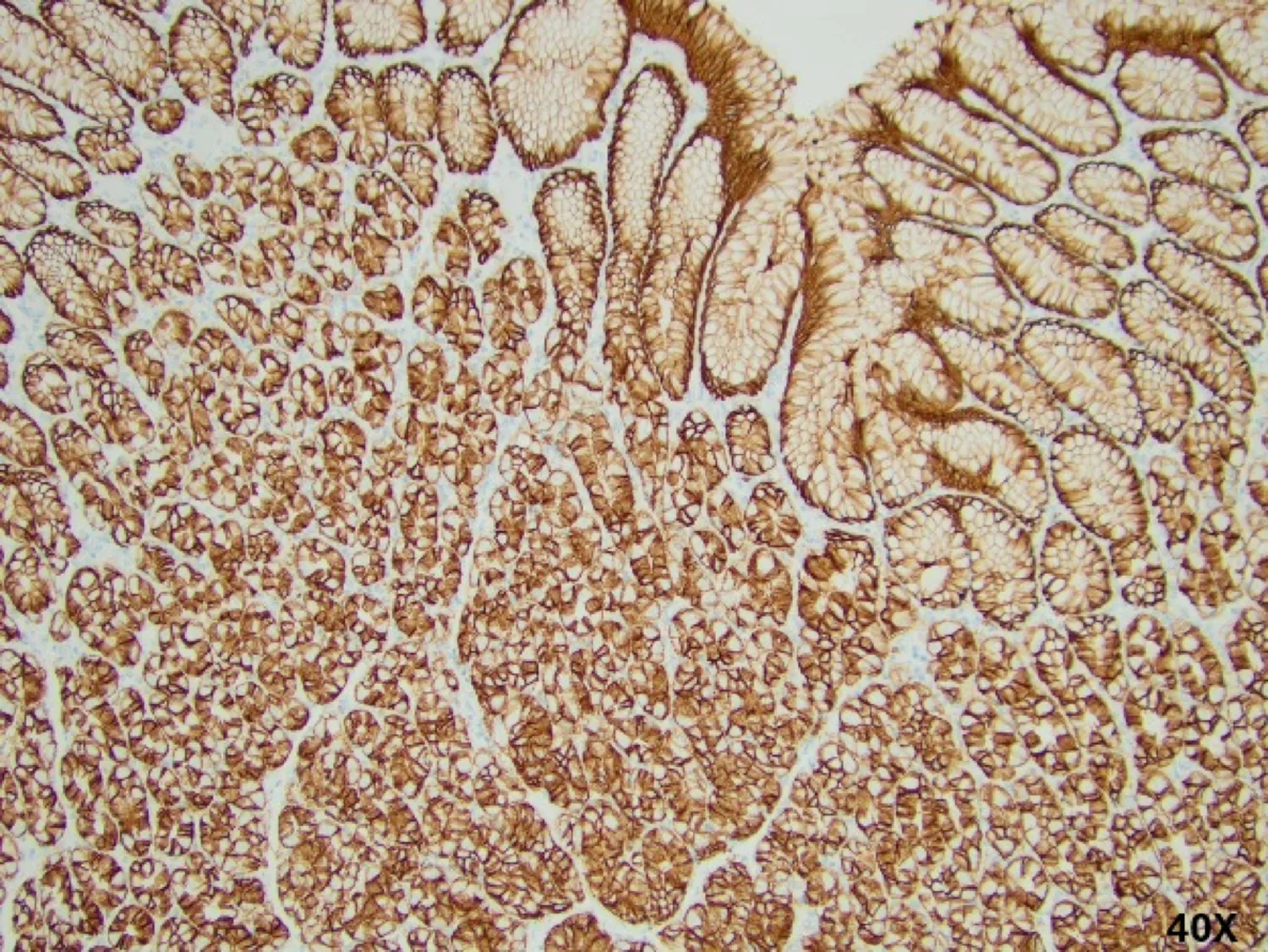
Positive control for Claudin-18 immunohistochemical stain on benign gastric epithelium (40X)

Claudin 18 immunohistochemical staining was performed on the most representative formalin-fixed, paraffin-embedded tumor tissue for each case. Normal gastric tissue was used as a positive control for each of the two staining batches performed, with expected staining shown in the controls (Fig. 1). All study slides met the laboratory’s staining-acceptability criteria.

The resulting slides were independently reviewed and interpreted by both investigators, who were blinded to the clinicopathologic data. Complete concordance was achieved with both investigators. We adopted the Claudin 18 IHC interpretation method described in the study by Brust et al. [9]. By this method, the intensity of staining was assigned a value of 0 (none), 1 (weak), 2 (moderate), or 3 (strong). Percentage of positive cells was assigned a value of 0 (0%), 1 (< 10%), 2 (10–50%), 3 (51–80%), or 4 (> 80%). The intensity score and percentage score are multiplied together to give a final IRS ranging from 0 (negative) to 12 (strongly positive).

## Results

Forty-one of the cases were from the parotid gland, four cases from the submandibular gland while the remaining three were from minor salivary glands (lip and palate). Within the mucoepidermoid carcinomas, seven were low grade, while two were of intermediate grade. The adenoid cystic carcinomas were predominantly low grade with tubular and cribriform architecture, with only one case comprised of solid architecture. The three cases of carcinoma ex-pleomorphic adenoma were salivary duct carcinomas.

**Fig. 2:**
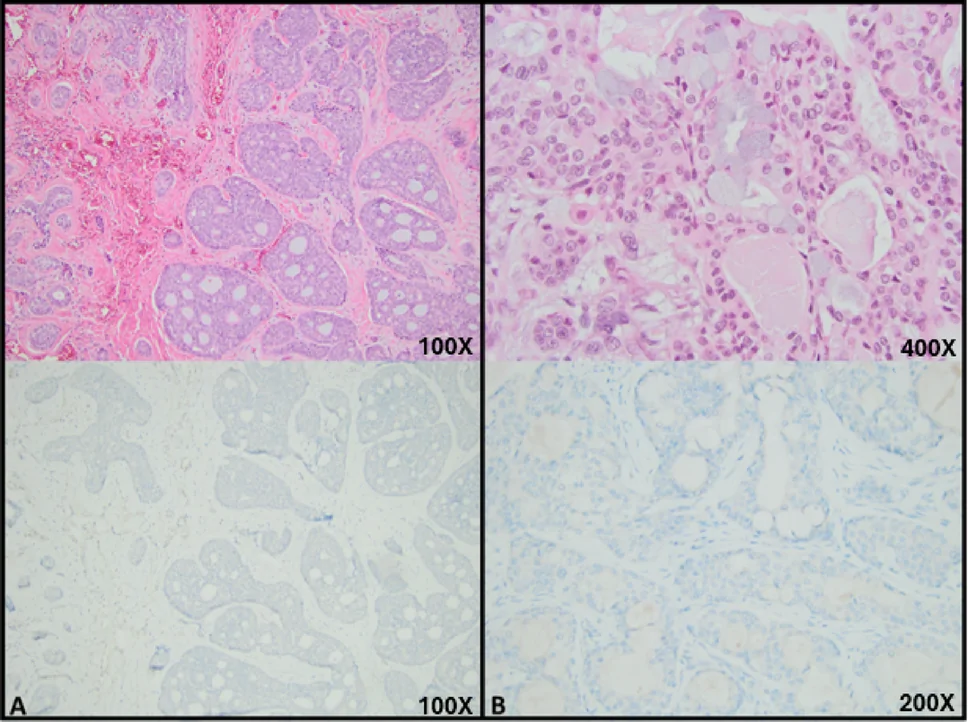
Representative H&E image with corresponding Claudin-18 immunohistochemical stain for adenoid cystic carcinoma. **A** Top left- H&E 100X, Bottom left- CLDN18 IHC 100X) and mucoepidermoid carcinoma. **B** Top right- H&E 400X, Bottom right- CLDN18 IHC 200X). H&E and associated IHC images are from serial sections of the same tumor block. All cases received an IRS of 0

Diffuse and strong membranous staining of Claudin 18 was observed in the non-neoplastic gastric epithelial control tissue (Fig. 1). In salivary gland tissue, Claudin 18 expression was absent in all benign and malignant neoplastic tissue. There was also no staining in the surrounding non-neoplastic salivary gland tissue (Fig. 2). All cases received an IRS of 0 (negative) (Table 2). One case of Warthin tumor demonstrated granular cytoplasmic staining in epithelial cells, and it was interpreted as negative. While the IRS was utilized to facilitate direct comparison with Brust et al. [9], the therapeutically relevant staining pattern for selection of patients eligible for Zolbetuximab is moderate to strong membranous staining in at least 75% of tumor cells [10]. Therefore, the overall clinical interpretation of this cohort would remain unchanged using the clinically validated companion-diagnostic criterion, as none of the cases demonstrated the required membranous staining pattern. The granular cytoplasmic staining observed in the Warthin tumor was likewise not relevant to the companion-diagnostic definition of positivity because the staining was non-membranous.

**Table 2:** The results of this study were uniformly negative (IRS = 0)

Salivary gland neoplasm	Staining intensity score	Percentage of positive cells score	Total IRS
Basal Cell Adenoma	0	0	0
Oncocytoma	0	0	0
Pleomorphic Adenoma	0	0	0
Warthin Tumor	0	0	0
Acinic Cell Carcinoma	0	0	0
Adenoid Cystic Carcinoma	0	0	0
Carcinoma ex-Pleomorphic Adenoma	0	0	0
Mucoepidermoid Carcinoma	0	0	0
Polymorphous Adenocarcinoma	0	0	0
Salivary Duct Carcinoma	0	0	0
Salivary Gland Carcinoma, Not Otherwise Specified	0	0	0

The breakdown of IRS is shown relative to tumor type in this table. The intensity of staining was assigned a value of 0 (none), 1 (weak), 2 (moderate), or 3 (strong). Percentage of positive cells was assigned a value of 0 (0%), 1 (< 10%), 2 (10–50%), 3 (51–80%), or 4 (> 80%). The intensity score and percentage score are multiplied together to give a final IRS ranging from 0 (negative) to 12 (strongly positive)

## Discussion

Salivary gland malignancies are rare entities that encompass 1–5% of all head and neck cancers. Treatment of these tumors varies by case, and recommended guidelines include a variety of surgical and systemic therapies [11]. In select cases, targeted therapy for biomarkers such as NTRK, HER2, and AR can be pursued, with positive outcomes [7].

Claudin proteins are directly involved in the integrity of tight junctions in organs with tubular differentiation. During embryogenesis, claudins 1, 3, 4, 5, 7, and 11 are expressed at various timepoints and are hypothesized to be involved in morphologic differentiation of mature salivary gland tissue [12]. In adults, claudins 1, 3, 4, 5, and 7 are known to be expressed in mucoepidermoid carcinomas [8].

Aberrant Claudin 18 expression has been documented across a diverse range of malignancies (pancreatic adenocarcinoma, cholangiocarcinoma, and ovarian mucinous carcinoma), extending its relevance well beyond gastric cancer [5]. One study focusing on Claudin 18 expression in gastrointestinal pathology noted positive staining in 75% of adenocarcinomas of the gastroesophageal junction and 68% of gastric adenocarcinomas [13]. Additionally, 80% of gastric neuroendocrine tumors expressed Claudin 18, and a significant portion of samples with intestinal metaplasia or high-grade dysplasia also demonstrated Claudin 18 expression. However, not all of these immunohistochemical staining patterns met criteria for inclusion in clinical trials (2+ or higher staining in at least 50% of lesional cells). Thus, even though there were not statistically significant differences in the expression of Claudin 18 amongst varying levels of dysplastic change within the gastrointestinal organs, the aberrancy of staining to qualify for inclusion to clinical trials was significantly higher in cases of high-grade dysplasia and adenocarcinoma [13].

Within pulmonary tissue, the expression of Claudin 18 varies between adenoid cystic carcinomas and mucoepidermoid carcinomas. One study utilizing H scores to quantify staining patterns noted that Claudin 18-positive adenoid cystic carcinomas had higher H values (and therefore greater intensity of staining and number of cells staining) than Claudin 18-positive mucoepidermoid carcinomas. H scores were also notably higher for Claudin 18 expression in tumors of patients with a smoking history than those without a smoking history [14].

The discovery of aberrant Claudin 18 expression in gastric malignancies has led to the advent of Zolbetuximab, an anti-Claudin 18.2 antibody, to be administered in combination with chemotherapy as a first-line treatment for patients with Claudin 18.2 expression in locally advanced unresectable gastric or gastro-esophageal carcinomas [15]. Phase 3 clinical trials have indicated that approximately 38.4% of unresectable or metastatic gastric and gastro-esophageal carcinomas meet the clinically validated Claudin 18.2 positivity criterion (moderate to strong membranous staining in at least 75% of tumor cells) [10]. Zolbetuximab in combination with chemotherapy demonstrates significant increase in overall survival and progression-free survival in these patients [16].

A search of the literature reveals one other study investigating the expression of Claudin 18.2 in salivary gland carcinomas. In that study, Claudin 18.2 was somewhat expressed in 6% of salivary gland carcinomas (including mucoepidermoid carcinoma, acinic cell carcinoma, and adenoid cystic carcinoma), but the immunoreactivity score (IRS) was less than 1, which was considered an overall negative phenotype. Claudin 18.2 expression was not significantly associated with higher tumor stage [9]. Our study similarly notes negative expression of Claudin 18 across a variety of salivary gland carcinomas, and negative expression was present irrespective of tumor location, grade, and stage. While these findings are consistent with prior reports [9] and provide independent confirmation, the present study extends the evaluation to a broader spectrum of benign and malignant neoplasms, further demonstrating the consistently limited expression of Claudin 18 across salivary gland entities.

This study hypothesized that due to the possible glandular or secretory nature of salivary glands, aberrant Claudin 18 expression may be present in salivary gland neoplasms and may serve as a biomarker for targeted therapy. Although none of the 48 cases evaluated in this sample demonstrated positive Claudin 18 staining (0%; 95% CI, 0–7.4%), the absence of positive cases in this limited cohort should not be interpreted as a true prevalence of 0%. Our findings suggest that Claudin 18 expression is rare in salivary gland neoplasms, but Claudin 18 expression in uncommon salivary gland tumor subtypes not evaluated in this sample cannot be excluded. However, the uniformly negative results across some of the more frequent salivary gland neoplasm subtypes add to the limited pre-existing salivary gland data suggesting that Claudin 18 is unlikely to serve as a viable therapeutic target in this organ.

One possible explanation for the lack of Claudin 18 expression is that salivary gland and gastric mucosa represent distinct differentiation programs despite both producing mucin. This is supported by the fact that the predominant mucin glycoproteins differ between these organs. Salivary glands primarily express MUC5B and MUC7, while gastric epithelium predominantly expresses MUC5AC and MUC6 [17]. This hypothesis is supported by the findings in study by Bian et al., in which Claudin 18 expression was significantly and positively corelated with MUC5AC and MUC6 expression in pulmonary invasive mucinous adenocarcinoma [18]. Although these findings are extrapolated from another organ system and this relationship was not directly evaluated in the present study, differences in epithelial phenotype may reflect underlying variations in lineage-specific gene expression and cellular differentiation pathways. Further evaluation of mucin programs in salivary gland tumors is needed to directly investigate this potential relationship. As Claudin 18 is strongly associated with gastric epithelial differentiation, the absence of expression in salivary gland neoplasms may indicate that salivary gland mucinous differentiation does not mimic the molecular programs that drive Claudin 18 expression in gastric tissues.

## Data Availability

Data is available upon request.

## Code Availability

No software application or custom code was used in preparation of this article.
